# Exploring a new method for the assessment of metal exposure by analysis of exhaled breath of welders

**DOI:** 10.1007/s00420-022-01833-z

**Published:** 2022-01-23

**Authors:** Göran Ljungkvist, Håkan Tinnerberg, Jakob Löndahl, Therese Klang, Emilia Viklund, Jeong-Lim Kim, Linus Schiöler, Niklas Forsgard, Anna-Carin Olin

**Affiliations:** 1 Occupational and Environmental Medicine, Department of Public Health and Community Medicine, Institute of Medicine, Sahlgrenska Academy, University of Gothenburg, Box 414, 405 30 Gothenburg, Sweden; 2 Division of Ergonomics and Aerosol Technology, Department of Design Sciences, Faculty of Engineering, Lund University, Lund, Sweden; 3 Sahlgrenska University Hospital, Gothenburg, Sweden

**Keywords:** Exhaled breath, Welding, Biomarkers, Metals, Exposure, Small airways

## Abstract

**Purpose:**

Air monitoring has been the accepted exposure assessment of toxic metals from, e.g., welding, but a method characterizing the actual dose delivered to the lungs would be preferable. Sampling of particles in exhaled breath can be used for the biomonitoring of both endogenous biomarkers and markers of exposure. We have explored a new method for the sampling of metals in exhaled breath from the small airways in a study on welders.

**Methods:**

Our method for particle sampling, Particles in Exhaled Air (PExA®), is based on particle counting and inertial impaction. We applied it on 19 stainless steel welders before and after a workday. In parallel, air monitoring of chromium, manganese and nickel was performed as well as blood sampling after work.

**Results:**

Despite substantial exposure to welding fumes, we were unable to show any significant change in the metal content of exhaled particles after, compared with before, exposure. However, the significance might be obscured by a substantial analytical background noise, due to metal background in the sampling media and possible contamination during sampling, as an increase in the median metal contents were indicated.

**Conclusions:**

If efforts to reduce background and contamination are successful, the PExA® method could be an important tool in the investigations of metals in exhaled breath, as the method collects particles from the small airways in contrast to other methods. In this paper, we discuss the discrepancy between our findings and results from studies, using the exhaled breath condensate (EBC) methodology.

## Introduction

Worldwide, it is estimated that 11 million workers have the job title of welder, and around 110 million more have job tasks with potential welding-related exposure (Antonini 2003). Welders are exposed to toxic gases and aerosols, which can cause bronchiolitis, bronchitis, airway irritation, pneumonitis (Antonini 2003) and asthma (Storaas et al. 2015). Most welding materials are alloy mixtures of metals, and of these, chromium, nickel and manganese are of particular interest from a toxicological point of view. Manganese exposure is associated with severe neurological diseases (Martin 2006), and chromium and nickel, as well as welding fumes per se, are classified as carcinogenic to humans (Group 1) by the International Agency for Research on Cancer (IARC) (Hopkins 1991; Guha et al. 2017).

The characteristics of welding fumes are complex and depend on several factors such as base materials and surface coating, the welding process, the filler material, fluxes, shield gas composition, spray type and voltage/current applied (see (Antonini 2003) for a review). Finally, the dose delivered to the welder is dependent on the technique and skill of the welder, workload, surrounding welding activities, degree of enclosure, general and local ventilation and the use of personal protection equipment (PPE) (Persoons et al. 2014; Pesch et al. 2012; Weiss et al. 2013).

During the welding process, the metals from the base material and filler evaporate because of the high temperature and then condense to particles. The primary particles are ultrafine, with a diameter of 0.01–0.10 µm, but owing to agglomeration, the primary particles will form chains and the resulting size distribution is multimodal and undergoes dynamic change over time. Splatter particles are also ejected during the welding process (Zimmer and Biswas 2001). Typically, the diameters of welding particles range from 0.1 µm to 1 µm. The chemical characteristics of welding particles regarding composition, oxidation states of the metals and solubility are complex (Zimmer et al. 2002; Taube 2013; Berlinger et al. 2011, 2019).

The ultimate goal of exposure assessment is to quantify the dose delivered to the target organ. This has till now not been practically possible for substances with lung toxic effects. The accepted standard for assessing exposure to most airborne toxic substances is personal monitoring in the breathing zone. Metal aerosols are collected on filters and subsequently analysed by atomic absorption (AA) or inductively coupled plasma mass spectrometry (ICP-MS). Different types of sampling devices, mostly cyclones, are used to selectively sample the respiratory fraction of the aerosol. There are several drawbacks with this methodology. As the welding aerosol forms a plume, the placement of the sampler is crucial and can bias the result. In addition, it does not account for the workload and thereby the inhaled air volume. Finally, the metal concentration is with few exceptions measured outside PPE and is, therefore, not representative of the inhaled fraction. Biological monitoring of metals in blood, and urine, is an alternative and e g the analysis of lead and cadmium in blood have reached regulative status in many countries. For chromium, manganese, and nickel biological monitoring may be suitable on group level but not on individual level (Smith et al. 2007; Gube et al. 2012; Weiss et al. 2013; Baker et al. 2014). Also, blood sampling is invasive and urine collection is more delicate from an integrity point of view. Furthermore, blood and urine reflect all intake routes of the substance, including food intake, which is a drawback if the focus is toxic effect in the lungs or respiratory uptake. Bronchoalveolar lavage (BAL) reflects a diluted sample of the respiratory tract lining fluid (RTLF), but it is invasive, takes time, must be performed in a medical setting and cannot easily be repeated in short intervals, e.g. before and after shift.

Exhaled breath has emerged as a promising matrix for biological monitoring. Particles, or droplets, are formed from RTLF during breathing and can subsequently be collected by condensation, filtration or impaction. It has none of the drawbacks of BAL mentioned above. The methodology was originally used in non-invasive biomonitoring of injury preceding clinical disease of the lung (Beale et al. 2017; Davis and Montpetit 2018), including biomonitoring of transition and post-transition metals. It has also been applied for exogenous compounds such as drugs (Beck et al. 2013) and for metals in industrial settings (for a review, see (Ghio et al. 2018)). Analysis of metals in exhaled breath has the potential to improve the evaluation of dose–effect relationship for substances toxic to the lung as it better reflect the actual dose delivered. Also, the contribution from inhalation can be assessed in cases where multiple intake routes are present. In a longer perspective it could also perhaps be used for regulative exposure assessments.

The full picture of the mechanisms involved in particle generation is still not clear. An accepted mechanism is generation during the reopening of the small airways after collapse during the previous exhalation (Johnson and Morawska 2009; Almstrand et al. 2010; Bake et al. 2019). Atomisation resulting from an airstream passing over the surface of a liquid with sufficient speed, preferably in the upper airways, is also suggested.

Until now all studies of metals in exhaled air have been performed using the exhaled breath condensate (EBC) methodology. Basically, the exhaled air is cooled to condensate, which is collected and analysed. Several different devices are commercially available. The collecting efficiency differs depending on cooling temperature and geometry of the device but estimates of about 50% recovery of the water content have been published (Gessner et al. 2001; Konstantinidi et al. 2015). The collecting efficiency of non-volatile components such as metals is, to our knowledge, less known. The particle generation mechanisms involved is unclear, beside a small contribution from airway opening at tidal breathing. However, the upper airways are suggested as site of formation, but evidence is weak (Bondesson et al. 2009; Davidsson et al. 2005; Marteus et al. 2005).

Our research group has developed a method for collecting particles in exhaled breath based on inertial impaction and counting of particles. The method, which is called the Particles in Exhaled Air (PExA®) method, is optimized to collect particles generated in the small airways during opening of the airways (Almstrand et al. 2009, 2010; Bake et al. 2017, 2019). The collected particles are referred to as “PEx” in the present paper.

In a pilot study (Bredberg et al. 2014), we exposed nine subjects to fumes from mild steel welding for 2 h. Manganese was chosen as the metal of interest and the exposure concentration was at the Swedish occupational exposure limit (OEL) of 50 µg/m^3^ (respirable dust). In four of the subjects, detectable amounts of manganese and iron in PEx were analysed directly after the exposure. After 24 h, no metal content was detected in PEx from any of the subjects. The results encouraged us to proceed with a limited study in an authentic welding environment.

The purpose of the present study was, first, to explore PExA® as a method for the assessment of toxic metals in welders. We extended the scope of the investigation, compared to the pilot study, to also cover chromium and nickel, in addition to manganese. Therefore, we chose a workplace with exclusive stainless steel welding. Second, we aimed to study the correlations between the metal content of PEx and current metal exposure in respirable air and cumulative exposure expressed as welding years. In parallel, we analysed the metal content of whole blood and plasma. Finally, in order to estimate exposure and validate the results, we modelled the deposition fraction and mass per surface area of welding particles in different generations of the respiratory tract.

## Methods

### Study population and design

Nineteen welders (18 men, one woman) were recruited from a plant manufacturing stainless steel tanks. Morphometric, demographic and employment data, smoking status and medical history were recorded. All welders underwent exposure measurements and a clinical examination including exhaled air analysis, lung function testing, blood sampling and the completion of questionnaires.

The welders answered a questionnaire regarding working history with emphasis on welding and other potential exposure to metals. Also, they completed a work diary starting from the preceding weekend and including the day of exposure measurement. The questionnaires were followed up with interviews conducted by a trained occupational hygienist. Nineteen technicians, care and laboratory staff with no occupational metal exposure, served as controls for the blood measurements.

The study was approved by the Ethical Review Board of the University of Gothenburg (registration No. 715-12) and met the principles of the Declaration of Helsinki. The participants gave their written informed consent to participate in the study.

### Description of the work facilities and processes

The plant where the welders worked manufactures steel tanks for transport of liquid gases. The cryogenic tanks were assembled in sections and some work activities were performed from inside the partly assembled tank. All welding was done in stainless steel, typically alloyed with 18–20% chromium, 8–12% nickel and 2% manganese. The filler materials had a similar composition.

A large variety of welding methods were used as follows: shielded metal arc welding (SMAW; or stick welding), gas metal arc welding (GMAW/MMA; also known as metal inert gas (MIG) or metal active gas (MAG) welding), flux-cored arc welding (FCAW), gas tungsten arc welding (GTAW; or tungsten inert gas (TIG) welding) and plasma cutting. A working day typically consisted of preparations, cutting, welding and grinding. Half of the welders used more than one welding technique during the day of exposure measurement. The mix of methods and work tasks, performed exclusively in stainless steel, constituted an adequate basis for the study, considering its exploratory purpose.

Most of the work at the plant was performed in two spacious large production halls with good general ventilation of displacement type. Local exhaust ventilation (LEV) was available in most welding situations. Not only different types of welding but also grinding were performed in the halls and there were no curtains to separate different working stations. Three of the welders worked in specially ventilated welding stations.

The workers’ PPE varied from an eye protection helmet (“welding helmet”) to filtering facepieces, half mask respirators and powered air-purifying respirators (PAPRs).

### Exposure measurements

Exposure measurements were performed during one full shift per welder on either Tuesday, Wednesday or Thursday to reflect full-shift work exposure.

To assess the respirable fraction of the welding aerosol, the welder wore a headset with a mini cyclone sampler (Lidén et al. 2014). The arrangement allowed measurement inside the eye protection helmet or the PAPR, but not inside filtering facepieces or half mask respirators. Battery-powered sampling pumps (SKC AirChek XR5000; SKC Inc., Eighty Four, PA, USA) were operated at a flow rate of 0.75 L/min. The pump airflows were verified at start and stop of each sampling period (pre- and post-lunch break).

### Measurement of particles in exhaled breath using the PExA® method

**Fig. 1:**
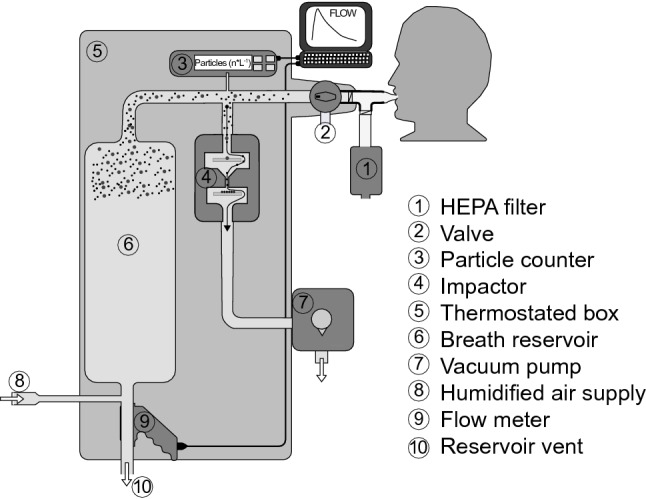
Schematic presentation of the PExA® instrument. HEPA = high-efficiency particulate air. Adopted from (Larsson et al. 2017)

The PExA® instrument has been described previously (Almstrand et al. 2009). A schematic presentation of the instrument is given in Fig. 1. Briefly, the subject exhaled through a double-valve system. The first valve was a two-way non-rebreathing valve (Hans Rudolph, Shawnee, KS, USA) which allowed the individual to inhale filtered air from the room (Whatman HEPA-CAP® filter; GE Healthcare, Little Chalfont, UK) and exhale either to waste or into the instrument by opening the second valve.

The method was optimized to collect particles from the small airways. Physiologically, the method was based on the formation of particles during the reopening of the small airways after collapse during the previous exhalation (Almstrand et al. 2010; Bake et al. 2019; Johnson and Morawska 2009). Therefore, a special breathing manoeuvre was used to maximize this formation of particles. The breathing manoeuvre started with an exhalation to residual volume, a breath hold for 5 s, a rapid inhalation to total lung capacity, followed by a deep exhalation at a spontaneous flow rate. By opening the second valve, only this final exhalation was collected by the PExA® instrument. The breath reservoir enabled the entire respiration to bypass the impactor.

**Fig. 2:**
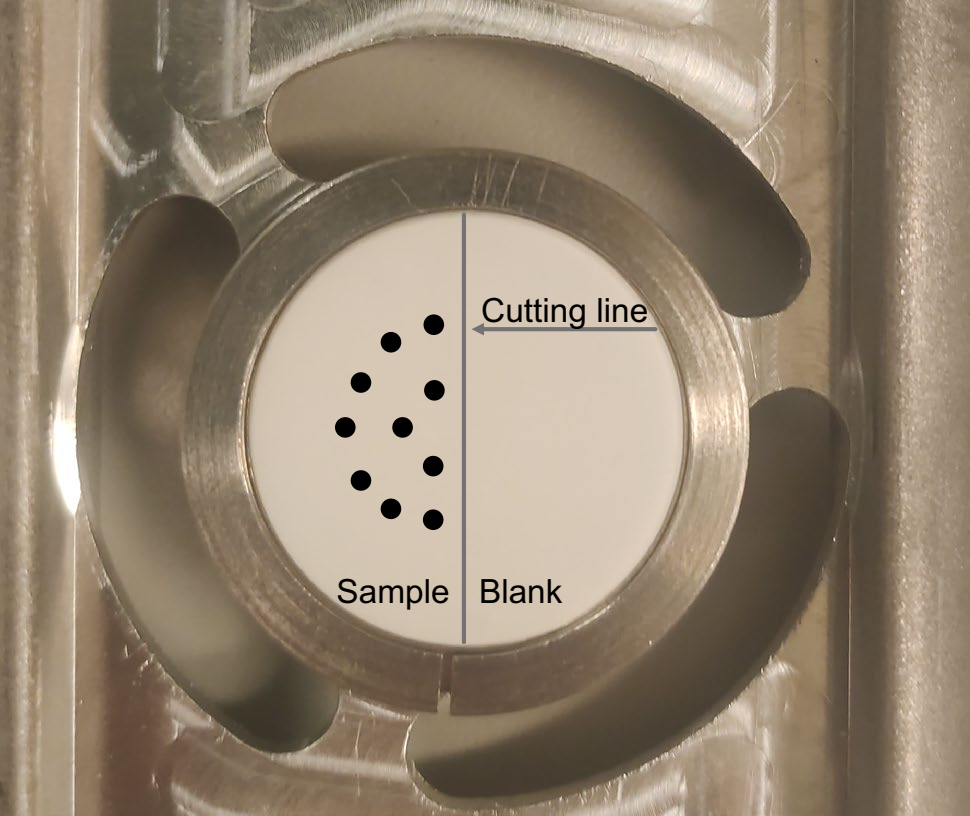
The collecting membrane. The exhaled particles are collected as spots corresponding to the impactor nozzles on the sampling half of the membrane. The other half is used as the corresponding blank

The PExA® instrument collects particles by the impaction principle. The impactor (PM10 Impactor, Dekati Ltd., Tampere, Finland) was designed to collect particles with an aerodynamic diameter of between 0.5 and 5 µm. During the collection, the particle concentrations were measured in eight size intervals by an optical particle counter (Grimm 1.108; Grimm Aerosol Technik GmbH, Ainring, Germany). From the particle counter measurement, the collected particle mass was estimated online by an in house developed software. In practice, about 85–95% of the collected particle mass consists of particles with a diameter < 2 µm (Bake et al. 2017). The particles impacted on a hydrophilic polytetrafluoroethylene (PTFE) membrane (Millipore FHLC 02500, Merck, Darmstadt, Germany). No air passes the membrane; it only serves as an inert surface for the particles to impact on. The impactor nozzles are located on one-half of the impaction membrane (Fig. 2) to facilitate a blank for each measurement.

After collection, the membrane was cut in two in a stream of HEPA filtered air, where the sample half and the corresponding blank half were placed in separate Eppendorf vials (pre-rinsed with 5% nitric acid and Milli-Q ultrapure water). The scalpel used was rinsed in ethanol before each cutting. Samples were stored at -20 °C. The amounts of metals in PEx were determined by subtracting the analysed amounts on the blank half of the membrane from the amounts on the corresponding sample half.

The instrument box and the tubing to the two-way valve were thermostated at 36 °C. Finally, the flow through the instrument was measured by a flow meter (OEM Flow Sensor Spiroson-AS; Medical Technologies, Zurich, Switzerland) that monitored flow velocities and exhaled volume. The mass of the collected particles was calculated using the information from the particle counter, assuming spherical particles and a density close to water.

The PEx samples were collected before and after the work shift on the same day the exposure measurements were performed.

### Lung function test

Spirometry was performed using a Spirare device (SPS3110 sensor) and Spirare 3 software (Diagnostica AS, Oslo, Norway) in accordance with current European Respiratory Society (ERS) and American Thoracic Society (ATS) standards (Miller et al. 2005). Spirometry data were expressed as percent predicted (% pred) based on local reference values from Brisman et al. (Brisman et al. 2016, 2017).

### Blood sampling

Blood samples from the welders were taken after shift on the day of the exposure measurement using a standard steel needle. Vials for trace metal analysis were used (BD Vacutainer cat No. 368361 ethylenediamine tetra-acetic acid (EDTA) vials; Becton & Dickinson, Franklin Lakes, NJ, USA). The first collected vial was discarded, and the two following were collected as samples. One vial was used for whole blood analysis. The other was centrifuged, and the plasma was transferred to another vial (Sahrstedt cat No. 72.694.007; Sarstedt Ag, Nümbrecht, Germany). The samples were stored at 4–5 °C before analysis.

### Metal component analysis

PEx samples were analysed by ICP-MS (Agilent 7700x; Agilent Technologies, Santa Clara, CA, USA) operated in the helium collision mode after 10 min of ultrasonic extraction with 300 µL 2% nitric acid. The instrument limit of detection (LOD) for the metals was in the order of a pg/sample. However, the method LOD, including sampling, sample handling, storage and analysis (based on the analysis of the blanks; *n* = 38, LOD = mean_blank_ + 3 × STD), was three orders of magnitude higher: 209, 634, and 426 pg/sample for chromium, manganese and nickel, respectively.

The filter samples from the aerosol monitoring were digested in a closed vessel with 5 mL 65% ultra-pure nitric acid, 0.25 mL 47–51% hydrofluoric acid and 1.5 mL 10% hydrogen peroxide in a microwave oven (Mars 5 microwave digester; CEM Corporation, Matthews, NC, USA). The samples were diluted with Milli-Q water to a nitric acid concentration of 1% and then further diluted with 1% nitric acid. Samples were analysed using an iCAP Q ICP-MS (Thermo Fisher Scientific, Waltham, MA, USA).

Whole blood and plasma samples were diluted 20 times with a basic diluent containing 1-butanol (2% w/v), EDTA (0.05% w/v), TritonX-100 (0.05%w/v) and ammonium hydroxide (1% w/v) prior to analysis on an Agilent 7700 × ICP-MS (Agilent Technologies, Santa Clara, CA, USA) operated in the helium collision mode.

### Deposition modelling

To estimate exposure and metal content on the lung surface, the deposition fraction and mass per surface area of welding particles in different generations of the respiratory tract were modelled. We used the Multiple Path Particle Dosimetry model, v. 3.04 (National Institute of Occupational Safety and Health, NIOSH, https://www.ara.com/mppd/), to obtain an estimate of the amount of welding particles that deposit in different regions of the respiratory tract (Miller et al. 2016). For the calculation, we used the Weibel model of the architecture of the lung and assumed oral breathing in an upright position at moderate breathing flows of 16 breaths per minute with a tidal volume of 1 L/min. Functional residual capacity was set to 3,500 mL, based on reference values for 40-year-old men of average height in Sweden (180 cm). For the welding aerosol, we assumed a size distribution with count median diameter of 0.16 µm, geometric standard deviation of 2.4 and a concentration of 1 mg/m^3^ (Isaxon et al. 2013; Zimmer 2002).

### Statistics

The continuous variables were presented as median with range or interquartile range (IQR) and the categorical variables as frequency (*n*). Differences in concentrations before versus after the exposure were tested using a non-parametric test (Wilcoxon signed-rank test), except for metals in plasma and whole blood, where Wilcoxon-Mann–Whitney test was used. The correlation between two quantitative variables was tested with the Spearman correlation test.

All statistical analyses were performed using version 9.3 of SAS for Windows (SAS Institute, Inc., Cary, NC, USA), applying two-tailed tests and a 5% level of significance.

## Results

### Study population

A summary of sex, age, smoking habits, total welding years and lung function is presented in Table 1. Lung function among the welders was considered as normal on average, but was found to be abnormal in some subjects. The subjects had a wide age range and reported a wide range of years of welding.

**Table 1:** Characteristics of the study population

Subjects, *N*	
Total	19
Men	18
Women	1
Age, years	48 (23–65)
Smoking status, *N*	
Never smokers	10
Ex-smokers	6
Present smokers	3
Years of welding	19 (3–39)
FVC, % pred	98 (72–114)
FEV_1_, % pred	91 (70–121)

Data are presented as median with minimum and maximum values

_*FVC* forced vital capacity, *FEV1* forced expiratory volume in 1 s_

### Metals in the collected particles

All samples, including the blanks, had quantifiable amounts of metals, according to the instrument LODs, but there was no statistically significant increase in the analysed metals in PEx over the working shift (*p* ≥ 0.6 for chromium, manganese, and nickel). However, the medians showed an increase over working shift, although a considerable statistical fluctuation, where blank values frequently exceeded the sample values. The results from the analyses, with the samples’ respective blanks subtracted, are summarized in Table 2. Just eight out of altogether 114 analysed metal concentrations in PEx exceeded the method LOD of the metal in question. We could not recognize any noticeable correlation pattern between these eight metal concentrations and collected PEx mass, smoking habits, lung function, exposure levels or long-term exposure (welding years).

**Table 2:** Medians and interquartile range (IQR) of the metal content in collected particle (PEx) samples from 19 stainless steel welders before and after exposure

Element	Before work shift (pg/sample)	After work shift (pg/sample)
	Median (IQR 25%–75%)	Median (IQR 25%–75%)
Chromium	4.3 (− 15.9 – 5.5)	6.1 (− 3.8 – 48.4)
Manganese	− 1.0 (− 31.5 – 105)	16.6 (− 41.2 – 77.8)
Nickel	− 3.3 (− 18.8 – 11.6)	4.4 (− 14.8 – 27.0)

The blanks were subtracted from the associated sample. Negative results are the consequence of blank values often exceeding the sample values

_*IQR* interquartile range_

### Exposure measurement

The sampling time was, on average, 6.0 h (range 4.2–7.0 h), lunch break not included. The median air concentrations of chromium, manganese and nickel are presented in Table 3. Fifteen subjects wore either an eye protection helmet or PAPR which enabled monitoring inside the device, therefore reflecting the actual exposure. Four subjects wore either a half mask respirator (*n* = 2) or a filtering facepiece (*n* = 2), preventing monitoring inside the mask. Generally, PPE was worn only during the actual welding sessions. In seven cases the Swedish OEL for manganese (respirable dust 50 µg/m^3^) was exceeded. There was no correlation between the metal content of PEx, and air concentrations or the accumulated exposure, expressed as welding years.

**Table 3:** Personal exposure to chromium, manganese and nickel of all welders, welders wearing either an eye protection helmet or PAPR (inside PPE, reflecting the inhaled air) and welders wearing a half mask or filtering piece (outside PPE, reflecting the air outside the PPE), presented as median and range

Element	All welders *n* = 19 (µg/m^3^)	Inside PPE *n* = 15 (µg/m^3^)	Outside PPE *n* = 4 (µg/m^3^)
	Median (range)	Median (range)	Median (range)
Chromium	5.5 (< 0.1 – 204)	4.9 (< 0.1 – 204)	6.2 (1.6 – 32.0)
Manganese	25.2 (< 0.1 – 648)	25.2 (< 0.1 – 648)	72.7 (1.6 – 156)
Nickel	2.2 (< 0.1 – 95.1)	3.3 (< 0.1 – 95.1)	1.7 (1.1 – 15.8)

_*PPE* personal protection equipment_

### Blood analysis

The levels of chromium, manganese and nickel in plasma and whole blood in workers and controls, respectively, are presented in Table 4. The levels of nickel in controls frequently fell below the LOD (0.16 µg/L) in both plasma (three out of 19 samples) and whole blood (13 out of 19 samples). In the statistical calculations, half the LOD (0.08 µg/L) was used in those cases (Hornung and Reed 1990). Levels of chromium and nickel from welders compared with controls were significantly elevated in both plasma (chromium *p* = 0.020, nickel *p* < 0.001) and whole blood (chromium and nickel *p* < 0.001).

**Table 4:** Median and interquartile range (IQR) of the levels of chromium, manganese and nickel in welders and controls, analysed in plasma and whole blood

Element	Welders (µg/L)	Controls (µg/L)
	Median (IQR 25%–75%)	Median (IQR 25%–75%)
Chromium plasma	0.93 (0.87 – 1.13)	0.76 (0.74 – 0.81)
Chromium whole blood	1.19 (1.11 – 1.39)	0.69 (0.74 – 0.79)
Manganese plasma	0.55 (0.53 – 0.64)	0.53 (0.46 – 0.62)
Manganese whole blood	7.88 (6.59 – 10.1)	8.75 (7.61 – 10.3)
Nickel plasma	0.36 (0.30 – 0.46)	0.19 (0.17 – 0.24)
Nickel whole blood	0.30 (0.23 – 0.34)	0.08 (0.08 – 0.19)

_*IQR* Interquartile range_

### Deposition modelling

**Fig. 3:**
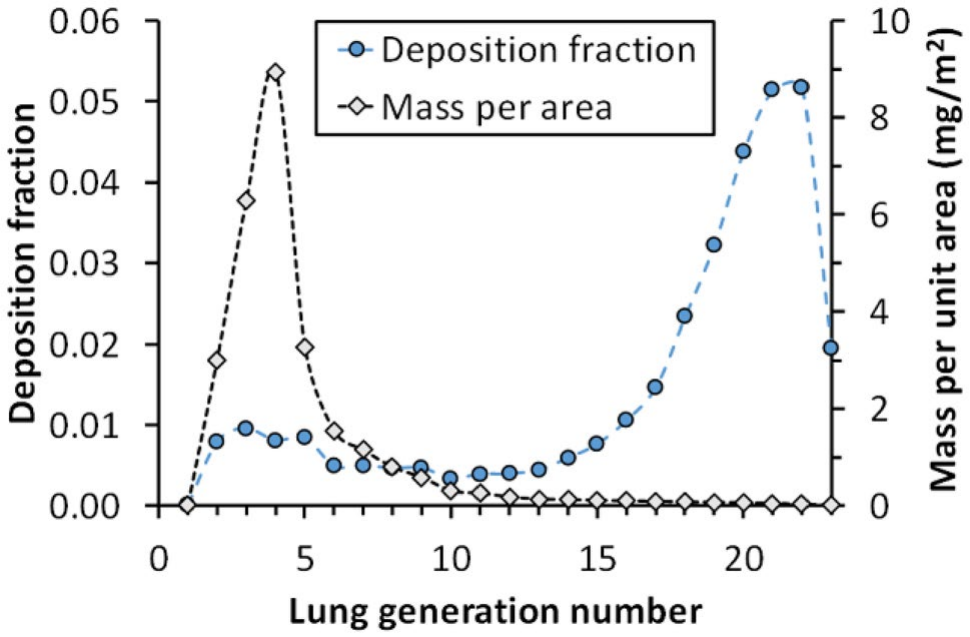
Deposition fraction and mass per surface area of welding particles in different generations of the respiratory tract. Although most particles deposit in the distal lung, below generation 10, the mass concentration is higher in the larger airways due to their relatively smaller surface area

The results of the deposition modelling are shown in Fig. 3. As expected, welding particles deposit in the distal lung and predominantly in the gas-exchange region of the airways (beginning at generation 15). However, the total surface area of the lungs in this region is about two orders of magnitude larger than for the conducting airways (generations 0–14). Therefore, the mass of deposited welding particles per surface area in the conducting airways, and especially the bronchi (generations 1–8), will be much higher.

## Discussion

We applied a method, PExA®, optimized for the collection of particles from the small airways, to assess exposure to chromium, manganese and nickel on stainless steel welders. We observed no significant increase in metal content in PEx over the working shift — despite the fact that air measurement as well as blood sampling, showed that the workers had substantial metal exposure. Consequently, there was no correlation between the individual air exposure or concentrations in blood and the amounts of metals in the corresponding PEx samples. However, the medians of the metal content in PEx samples were higher after shift compared to before for both chromium, manganese and nickel. This indicate that an increased metal content might be obscured by the substantial analytical background noise, where the blank part of the collecting media frequently exceeded the metal content of the sample part. As stated in the method description, the method LODs, including sampling and analysis, was higher by magnitudes compared with the instrument LODs. The reasons for this are partly the metal content of the sampling media (the PTFE membrane) and partly a possible contamination during the handling of the membrane before and after the actual sampling procedure. In our previously reported chamber exposure study (Bredberg et al. 2014), where we had access to laboratory facilities and a ventilated hood for sample handling, we achieved a method LOD (based on field blank analyses) for manganese that was somewhat lower, 450 ng/sample compared with 634 ng/sample in the present study. Most of the manganese derived from the blank membranes. The content of manganese differed from batch to batch and from filter to filter. To circumvent the problem in the present study, we cut the filters in two, where the exhaled particles were collected on one half and where the other half constituted an individual field blank. Even so, we were unable to find a significant difference between the metal content of sample and blank halves. The probable reason was contamination during sample handling. Sample collection was indeed performed in a facility apart from the manufacturing areas, but the participants came directly from work without changing from their work clothes, and without washing or showering. As the sampling medium was handled openly for a short while when the filters were cut and put into vials, dust from clothes and hair was a potential source of contamination of the collecting membrane.

The risk of contamination in field settings is substantial when analysing ubiquitous metals such as chromium, manganese and nickel in minute amounts and the history of metal biomonitoring is full of examples of this. Comparisons of LODs in studies performed using the ECB methodology are difficult as the results are mostly presented as metal concentration in the condensate. One possible approach is to compare the LODs in terms of concentration of metal in exhaled air. When using the reported instrument Limit of Quantifications (LOQ), the median condensate volume and the volume of exhaled air, the equivalent LOQ in exhaled air was 8.8, 0.9 and 3.5 pg/L exhaled air for chromium, manganese, and nickel, respectively, in a study performed by Hulo et al. (Hulo et al. 2014). Hoffmeyer et al. (Hoffmeyer et al. 2011) reported instrument LODs, and calculated LODs in exhaled air were approximately 2 ng/L for chromium and 8 pg/L for nickel. If we divide our method LODs by the median volume of exhaled air from our subjects, we get 3.0, 9.6 and 6.5 pg/L exhaled air for chromium, manganese and nickel, respectively. In conclusion, our LODs, presented as the metal concentration in exhaled air, are of the same magnitude as the LOD/LOQs achieved by these two research groups. To our knowledge, no attempts to perform blank tests during field studies has previously been reported.

The LODs we obtained were not sufficient for exposure assessment of the welders at individual level. An indicated increase in metal content over shift might be obscured by the analytical background noise. To find a sampling media with lower and more uniform metal background is a priority. So forth, we have examined several filter media suitable for the impactor but found nothing better than the standard PTFE filter. A possibility is to use a variant of a new membrane technique where the impaction spots are punched out, PEx sPOT, which has recently been developed. Each impaction spot (*n* = 10) has a diameter of 1.2 mm, which would reduce the desorbed area of the membrane by a factor 20 and thus the background to the same degree. But an even more urgent measure is to reduce contamination during the sample collection. First step is to find a facility as free from contamination as possible. A second is to avoid contamination from the workers themselves and do the sample collection after changing from working clothes and if possible after washing and showering. A third step is to reduce open handling of the membranes and perform the cutting/punching of the membrane in a laboratory setting, preferably in a LAF bench.

From these experiences, we strongly advocate a thorough step-by-step examination of possible contamination sources in the laboratory setting. As example, we examined the membrane cutting using a scalpel and did not find any noticeable contribution. If possible, the aim should be to achieve LODs permitting stable analysis of samples collected from subjects without occupational exposure. But the far most important point is to perform field blank testing, covering all steps from the preparations for the collecting, the actual sampling procedure, handling and transport of sampling, storage and finally the work-up and instrumental analysis. Based on this procedure, realistic method LOD/LOQs should be evaluated, preferably expressed per volume exhaled air to enable comparison between different methodologies.

Even if the results were disappointing from a contamination point of view, it is worth to discuss the fact that we could not identify any significant difference between samples collected before and after exposure. If we conservatively use our method LODs, we end up with 3.5, 9.6 and 6.5 pg/L in exhaled air for chromium, manganese and nickel, respectively. Hulo et al. (Hulo et al. 2014) reported 83 pg/L and 18 pg/L, respectively, of manganese and nickel in the exhaled breath of workers performing mild steel welding (conversion calculations as above). Hoffmeyer et al. (Hoffmeyer et al. 2011) reported nickel in the order of 18 pg/L in the exhaled breath of workers performing stainless/mild steel welding. In conclusion, both groups reported increased levels of exhaled metals after exposure to welding fumes, levels that would have been possible to quantify by the PExA® method, despite the method LODs we achieved in our study population.

There are two crucial differences between the EBC methodology and the PExA® method, which should be discussed to explain these discrepancies. First, the EBC methodology does not intentionally discriminate large particles as PExA® does. Probably large particles (> 5 µm) can deposit in both types of instruments on the way to the collecting device, but the impactor in the PExA® equipment intentionally discriminates large particles. As the volume (weight) increases by the cube of the radius of the particle, even a few large particles can contribute substantially to the total mass. For instance, the volume of a single particle with a diameter of 5 µm has 1,000 times the volume of a 0.5-µm particle.

A second major difference is the breathing pattern applied during sampling. According to the recommendations from the ERS (Horvath et al. 2017), tidal breathing is used in the EBC methodology. On the other hand, in the PExA® method a specially designed breathing pattern is used to optimize the generation of particles in the small airways by the airway reopening mechanism. Hulo et al. (Hulo et al. 2014) speculate whether this particle-generating mechanism may contribute to the metal content in exhaled breath collected by EBC. However, according to our experience, only minor amounts of particles are formed by this mechanism during tidal breathing; about 5 ng during a normal EBC session of 15 min, as compared to 120 ng collected during a normal PExA® measurement.

It has been suggested that the EBC methodology collects particles mainly from the upper airways, even if evidence is weak (Bondesson et al. 2009; Davidsson et al. 2005; Marteus et al. 2005). However, we compared the collection of methadone by PExA® and the SensAbues filtration device (SensAbues AB, Huddinge, Sweden) and found that the amounts of methadone collected by the latter method were at least two orders of magnitude higher (Ljungkvist et al. 2017). Even if SensAbues is based on filtration, not condensation, a common feature with the EBC methodology is that neither of them discriminates larger particles, in contrast to the PExA® method. Our explanation for this discrepancy was that SensAbues collects larger particles generated in the upper airways and that the site of particle formation is of great importance. As illustrated in Fig. 3, most welding particles deposit in the distal lung and predominantly in the gas exchange region (beginning at generation 15), but the mass of deposited welding particles per surface area in the conducting airways, and especially the bronchi (generation 1–8), is much higher. Hence, exhaled endogenous particles that are generated from the distal parts of the lungs (for instance after airway closure and reopening) may contain lower amounts of welding particles as compared with particles generated in the conducting airways. Shear forces induced by high air velocities have for long been suggested as a mechanism for particle formation in the airways. In the distal airways the air velocities are much too low for such a mechanism, but the velocities are higher in the conducting airways, which may contribute to disposing of once deposited particles. This could also be a favorable factor for the EBC methodology as compared with PExA®. To conclude, a conceivable explanation for the difference between EBC methodology and the PExA® method could be that EBC collects larger particles with high metal loadings from the upper airways. These different collecting mechanisms should be investigated in terms of their intrinsic abilities to reflect both exposure and effects.

When scrutinizing the potential of biomonitoring of metals in exhaled breath, the physicochemical properties of the inhaled metal particles are also of great importance. As stated above, welding particles will deposit along the whole respiratory tract (Cena et al. 2014). The dominating fraction of particles deposited in the tracheobronchial airways will be phagocytized by macrophages, translocated towards the larynx and swallowed within hours. Particles that deposit in areas without mucus coverage may explain possible long-term retention and their possible detection in BAL (Geiser and Kreyling 2010). Once they have reached the alveoli, the clearance of sub-micrometer particles is extremely slow, with a half-life of 700 days in humans, and probably even longer for nanoparticles (Rinaldo et al. 2015). Also, insoluble compounds of iron, chromium and nickel, for example, have been shown to be stored in lung tissue (Ghio et al. 2018; Kollmeier et al. 1985). A minor fraction of particles phagocytized by macrophages in the alveoli is transported out and cleared by the mucociliarly stairway (Schmid et al. 2009). Experimental support of the physicochemical properties of metals in exhaled breath is scarce, but nanoparticles from welding have been identified in EBC (Fireman et al. 2008; Gschwind et al. 2016; Klein et al. 2020). In our previous chamber exposure study, four out of nine subjects had detectable metal concentrations in PEx and had a similar quote between manganese and iron, indicating that welding particles per se were collected and analysed. Our interpretation of the results from this study, considering what we know now, is that random particles from the very recent exposure (collection within 5 min) were dispersed from the airways and collected.

Furthermore, solubility is crucial for the fate and toxicity of inhaled metal compounds (Forest et al. 2017) and will vary from highly soluble to insoluble in physiological liquids (Berlinger et al. 2011, 2019; Taube 2013; Zimmer 2002). Metals dissolved in the RTLF will probably be rapidly resorbed but could theoretically be included in droplets formed by airway opening or other particle generation mechanisms. This could also apply to dissolved metal compounds from particles deposited in the alveolus and subsequently translocated to the bronchioles by excess surfactant. Hoffmeyer et al. (Hoffmeyer et al. 2012) reported a significant correlation between cumulative iron-exposure (welding years) and iron in EBC in welders “usually refraining from using a particular respirator (mask)”. Also, Hulo et al. (Hulo et al. 2014) estimated an individual cumulative exposure based on a weekly exposure index and years of welding and correlated it with the metals studied (manganese, nickel, iron and chromium). They observed a relationship between this cumulative index and manganese and nickel in EBC. If these observations are valid, it opens for an interesting possibility to assess long-term exposure to metals. A hypothetical interpretation is that these results reflect metals deposited in the very peripheral airspaces where the clearance is extremely slow. If so, a possible assessment strategy would be to sample the exposed workers after a leave, long enough to circumvent the influence from recent exposure.

## Conclusions

Determination of particles in exhaled breath is a potential matrix for the assessment of exposure to metals toxic to the lung, but several issues must be solved before it can be used for dose–response assessments or as a standard exposure indicator. Deposition and dispersion mechanisms of inhaled particles must be clarified, as must also the fate of the welding particles and its content in both the short and the long term. Finally, the impact of different collection methodologies, having different collection characteristics, needs to be investigated. In this study, we did not find a significant increase in metal content after, compared with before, a substantial exposure during a workday. Comparative studies between different methodologies are advocated. Still, the PExA® method could be an important tool, especially once the technical shortcomings of contamination are solved, for increasing our knowledge about metals in exhaled breath as the method collects particles predominately from the small airways. Of special interest is the possible reflection of long-term exposure that has been reported, as well as the use of metals as biomarkers of clinical disease. Although comparison between metals in exhaled air and metals in inspired air is a natural step in an initial phase of method development, the relation to markers of effect would of course be more relevant in testing the usefulness of the methodology.

## Data Availability

Relevant data are within the manuscript.

## Code availability

Statistical calculations and deposition simulations were performed using general software applications (SAS Inc. and NIOSH respectively). Calculations of PEx mass was performed by an online in-house developed software.
